# Butyric Acid Increases the Therapeutic Effect of EHLJ7 on Ulcerative Colitis by Inhibiting JAK2/STAT3/SOCS1 Signaling Pathway

**DOI:** 10.3389/fphar.2019.01553

**Published:** 2020-01-22

**Authors:** Xiaonan Tang, Xiang Li, Yufei Wang, ZhiHui Zhang, AnJun Deng, WenJie Wang, Haijing Zhang, Hailin Qin, LianQiu Wu

**Affiliations:** Laboratory of Bioactive Substances and Functions of Natural Medicines, Institute of Materia Medica, Chinese Academy of Medical Sciences and Peking Union Medical College, Beijing, China

**Keywords:** ulcerative colitis, butyric acid, JAK2, STAT3, SOCS1

## Abstract

Ulcerative colitis (UC) is a refractory chronic disease characterized by bloody diarrhea and mucosal or submucosal ulcers. There is an urgent need of new drugs for the treatment of ulcerative colitis. EHLJ7 is a quaternary coptisine derivative. Herein, we explored the therapeutic effect of EHLJ7 on dextran sodium sulfate (DSS)-induced ulcerative colitis (UC) in mice. Results showed that EHLJ7 have good effects on DSS-induced colitis. EHLJ7 significantly improved symptoms induced by DSS including of weight loss, colon contracture, disease activity index (DAI), inflammatory infiltration, and so on. Furthermore, results showed that EHLJ7 could enhance short-chain fatty acids (SCFAs) production especially butyric acid, suggesting that EHLJ7 could improve the metabolic disorder of intestinal flora to a certain extent. Further study indicated that EHLJ7 could cooperate with butyrate to exert its anti-ulcerative colitis effect by inhibiting the activation of janus kinase 2 (JAK2)/signal transducer and activator of transcription 3 (STAT3)/suppressor of cytokine signaling 1 (SOCS1) pathway. Therefore, EHLJ7 has a potential to be developed as a candidate for the treatment of colitis.

## Introduction

Ulcerative colitis (UC) is a non-specific chronic inflammatory bowel disease with diarrhea, abdominal pain, and bloody stool as the main clinical symptoms (Cooper et al., 1993; Pohjonen et al., 2019; Nishida et al., 2019). The lesions of UC are mainly in the mucosa or submucosa of the rectum and colon (Barros et al., 2019; Saw et al., 2019). In recent years, many clinical studies have confirmed that intestinal flora imbalance is closely related to ulcerative colitis (Xavier, 2016; Walujkar et al., 2018; Khan et al., 2019). In patients with ulcerative colitis, the numbers of beneficial bacteria in the intestines reduced, while the numbers of pathogenic bacteria and harmful bacteria increased (Nagao-Kitamoto and Kamada, 2017). Furthermore, studies found that the expression of short-chain fatty acids (SCFAs) in the intestinal flora of UC patients was significantly lower than that of normal people (Pohjonen et al., 2019; Zhuang et al., 2019; Danilova et al., 2019).

The SCFAs in mammalian gastrointestinal tract are produced by the digestion of carbohydrates by colonic anaerobic bacteria, mainly including acetic acid, propionic acid, and butyric acid (D’Argenio and Mazzacca, 1999; Niccolai et al., 2019). SCFAs not only serve as the main source of energy for intestinal mucosal cells, but also reduce the production of pro-inflammatory factors and reduce the incidence of colonic inflammation (Feng et al., 2018). Studies have found that butyric acid can enhance the intestinal mucosal immune barrier, thereby preventing bacteria and their metabolites from entering blood and causing inflammation (Jirsova et al., 2019; Schulthess et al., 2019).

Janus kinase 2 (JAK2) is a non-receptor tyrosine protein which is widely distributed in various tissues and cells and can be activated by various cytokines (Roskoski, 2016; Hubbard, 2017). JAK2 is signal transducer and activator of transcription 3 (STAT3). STAT3 exists in the cytoplasm, which is coupled with tyrosine phosphorylated by the upstream signal molecules and directly links the expression of extracellular signals and intracellular genes such as suppressor of cytokine signaling 1 (SOCS1) (Yang et al., 2015).

EHLJ7 is a monomeric compound derived from Quaternary coptisine and synthesized by our institute. Our previous studies have found that the Quaternary coptisine derivatives have a significant therapeutic effect on dextran sodium sulfate (DSS)-induced ulcerative colitis in mice (Zhang et al., 2017a; Zhang et al., 2017b). In this paper, we first explored the therapeutic effect of EHLJ7 on DSS-induced ulcerative colitis in mice, and examined the changes in the content of SCFAs in the intestine of mice after administration. Then we investigated the mechanism how EHLJ7 increases the level of SCFAs especially butyric acid to enhance the therapeutic effect on UC through the JAK2/STAT3/SOCS1 signaling pathway.

## Materials and Methods

### Reagents

**Figure 1:**
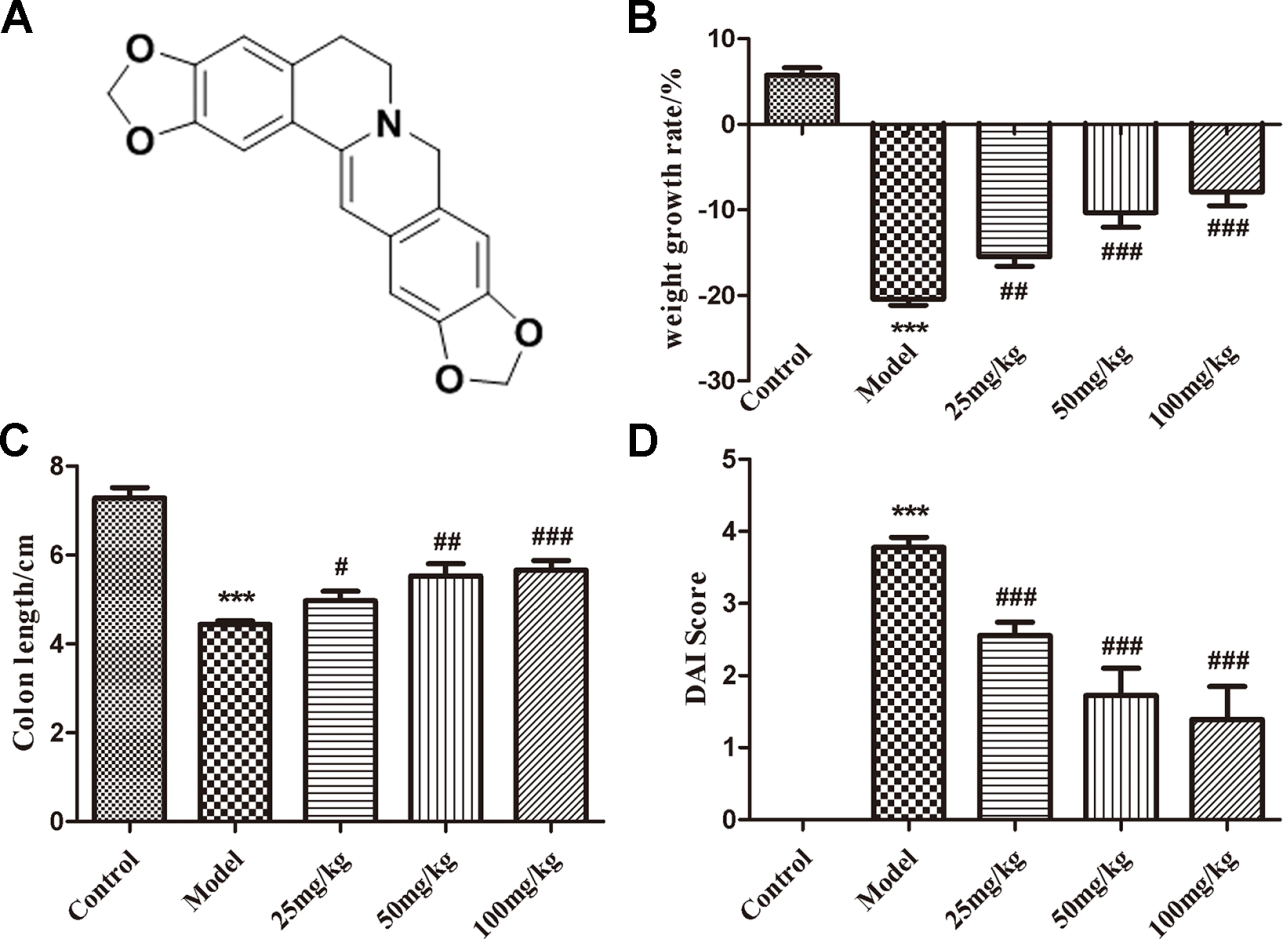
The structure of EHLJ7 and effects of EHLJ7 on weight growth rate, colon length, and DAI in DSS-induced UC mice. **(A)** The structure of EHLJ7. **(B)** EHLJ7 treatment (25, 50, and 100 mg/kg) effectively decreased UC related weight loss on day 7. **(C)** EHLJ7 treatment resulted in significantly longer colon length compared with DSS-only models. **(D)** EHLJ7 (25, 50, and 100 mg/kg) significantly reduced DAI score in mice. ***P < 0.001 compared with control group; ^#^P< 0.05, ^##^P < 0.01, ^###^P < 0.001 compared with DSS model group. Mean values ± SEM are shown. n = 6.

EHLJ7 was synthesized by our institute. The structure of EHLJ7 is showed in **Figure 1A**. Roswell Park Memorial Institute (RPMI) 1640 Medium and Hyclone™ fetal bovine serum (FBS) were both purchased from GE Healthcare (Waltham, USA). Interleukin-6 (IL-6) was obtained from Proteintech Group, Inc. (Chicago, USA). Sodium butyrate (SB) was purchased from Sigma Aldrich (St. Shanghai, China). Anti-JAK2, anti-p-JAK2, anti-STAT3, anti-p-STAT3, and anti-SOCS1 antibodies were purchased from Abcam, Inc. (Shanghai, China). DSS was obtained from MP Biomedicals (USA). Radio-immunoprecipitation assay (RIPA) lysis buffer was purchased from Sorlarbio Bioscience & Technology CO. INC (Beijing, China). Bicinchoninic Acid (BCA) Protein Assay Kit was purchased from Applygen Technologies Inc (Beijing, China). The ELISA kits for tumor necrosis factor alpha (TNFα) and IL-6 were from R&D Systems, Inc. (Minneapolis, USA). RNAprep pure Cell/Bacteria Kit, FastKing RT Kit, and SuperReal PreMix Plus were purchased from Tiangen Biotech (Beijing, China).

### Cell Culture

Intestinal epithelial cells IEC6 were obtained from the American Type Culture Collection (ATCC) and maintained in RPMI 1640 medium supplemented with 10% fetal bovine serum (FBS) at 37°C in a humid atmosphere with 5% CO_2_.

### Animal Experiment

Male C57BL/6J mice (18–20 g) were obtained from Beijing Huafukang Bioscience Co. Inc. (Beijing, China). Animals were adapted for feeding for a week in controlled conditions (25°C, 55% humidity, 12 h light/dark cycle). The mice in model and drug-administered groups were fed with 2.3% DSS (w/v) *via* drinking water for 7 days, and the mice in control group were fed with normal saline (n = 6). The mice in the three drug-administered groups were given different doses of EHL7 (25, 50, and 100 mg/kg) by oral gavage per day. On day 7, the animals in the model group showed obvious UC typical lesions such as decreased activity, weight loss, loose stools, and blood stools, indicative of the success of the model construction. Animals were then sacrificed and the colons were removed under a sterile environment. The contents in colon were frozen and stored in liquid nitrogen for SCFA detection. The colons were divided into three parts: one was fixed in 4% formalin for H&E staining and immunohistochemical assays, another was stored at -80°C for cytokine analysis, and the rest was used for Western blot analysis.

### H&E Staining and Immunohistochemical Assays

H&E staining was used to observe the pathological changes in the colons of UC model mice after DSS induction. Histopathological index (HI) was evaluated based on colon histopathology scoring criteria. The histological score was assessed as follows: the sections were graded using a range of 0 to 3 for epithelial injury and depth of ulceration, a range of 0 to 3 for edema, a range of 0 to 3 for infiltration (lymphocytes, monocytes, and plasmocytes) and depth of infiltration, a range of 0 to 3 for infiltration with neutrophils, and a range of 0 to 3 for infiltration with eosinophils and infiltration depth (Zheng et al., 2017). Immunohistochemical assays were carried out on 5 μm thick colon sections with anti-p-STAT3 and anti-SOCS1 antibodies diluted at 1:100.

### Detection of SCFAs Content in Feces

Fecal SCFA detection was carried out by BioNovoGene (Suzhou, China). The samples were thawed on ice and accurately measured 100 mg of feces for use. NaOH (0.005 M) aqueous solution and DL-2-methylbutyric acid were added and mixed. The mixtures were centrifuged and the supernatants were harvested, and 500 µL isopropanol/pyridine solution (3:2, v/v) and 100 µL propylchloroformate solution were added to the supernatants and mixed for 30 s. Then 300 µl n-hexane was added, mixed, and centrifuged, and the supernatants were harvested again. The two extracted supernatants and 10 mg of anhydrous sodium sulfate were mixed and centrifuged, and the supernatants were harvested again for gas chromatography–mass spectrometry (GC-MS) detection.

For the GC-MS study, Agilent 7890A/5975C instrument was used with Agilent HP-5 column (30 m*0.25 mm ID*0.25 μm) chromatographic column. The GC separation was carried out as follows: the inlet temperature was 250°C and ion source temperature 230°C. The transmission line temperature 250°C and quadrupole pole temperature 150°C. The starting temperature of the program was 60°C for 5 min and then was heated up to 110°C at 10°C/min. Then the temperature was heated up to 250°C for 1 min from 35°C/min temperature. The carrier gas is helium and the carrier gas flow rate was 1.0 ml/min. The total runtime was 15 min. After GC separation, analytes were ionized in EI. The MS acquisition was performed in selected ion monitoring (SIM) scanning modes with electron energy 70 eV.

### Tissue/Cell Lysis Preparation and Western Blot Analysis

RIPA lysis buffer was added to the collected tissues or cells, and incubated on ice for 30 min. BCA Protein Assay Kit was used to quantify the total protein of the supernatant. The supernatant was diluted with loading buffer and boiled at 100°C for 5 min. The expression of intracellular factors was detected using Western blot assay.

### ELISA Assays

Colon tissues were weighed, immediately frozen, and thawed three times in liquid nitrogen. Then, the samples were homogenized in lysis buffer (50 mM Tris-HCl pH 8.0, 150 mM NaCl, 1 mM ethylenediaminetetraacetic acid, 0.5% Triton X-100, and protease inhibitor). The colon homogenates were centrifuged at 10,000 ×g at 4°C for 20 min, and the supernatants were collected. The protein concentration of the supernatants were quantified using a BCA assay kit. TNFα and IL-6 levels were measured according to the R&D kit instructions.

### RNA Extraction and Quantitative Real-Time PCR

Total RNA was isolated from colon tissues using an RNAprep pure Cell/Bacteria Kit according to the manufacturer’s instructions. RT-qPCR was carried out using a FastKing RT Kit and SuperReal PreMix Plus. The sequences of primers of SOCS1, and glyceraldehyde 3-phosphate dehydrogenase (GADPH) were listed in **Table 1**.The reaction conditions were as follows: reverse transcription at 50°C for 30 min, followed by 35 cycles of pre-denaturation at 95°C for 2 min, denaturation at 94°C for 20 s, annealing at 58°C for 20 s, and extension at 68°C for 20 s. The relative mRNA expression was analyzed by 2^−ΔΔCt^ methods.

**Table 1:** Primers used for q-PCR analysis.

Type	Primer sequence
SOCS1	F: 5′-AACTCCCATTCCTCCACCTT-3′ R: 5′-GAGGGCCTCTCTCTTGCTCT-3′
GADPH	F: 5′-CCGCTCCCACTCTGATTACCG-3′ R: 5′-CGAAGCCATCTTCACGCTGA-3′

### Statistical Analysis

All data were calculated and are expressed as means ± SEM. T-test statistical method was used to analyze the significant difference between the two groups. Differences between groups were analyzed using either unpaired two-tailed Student’s t-test or one-way ANOVA with Bonferroni correction. P < 0.05 was considered statistically significant.

## Results

### EHLJ7 Attenuates DSS-Induced Body Weight Loss and Colon Contracture

EHLJ7 dose-dependently reduced body weight loss (**Figure 1B**) and inhibited colon contracture (**Figure 1C**) in mice induced by DSS. The model group mice had a weight loss of 20.43% while the administered groups of 15.48, 10.30, and 7.90% at doses of 25, 50, and 100 mg/kg, suggesting that EHLJ7 had a good effect on the weight loss of DSS-induced mice. In addition, the colonic contracture of model group mice compared with the control group was 39.13%, while those of the administered groups were 31.81, 24.03, and 22.20 respectively, suggesting that EHLJ7 induced a significant improvement in weight loss and colonic contracture of DSS-induced mice.

### EHLJ7 Attenuates the Increase in Disease Activity Index Score in UC Mice

Disease activity index (DAI) score is evaluated from indicators such as weight loss, loose stools, and blood stools (Cooper et al., 1993). The lower the DAI score is, the closer the animal is to the normal physiological state (The DAI scoring standard is shown as **Table 2**). Compared with the control group, the disease activity index (DAI) of the model group was significantly increased, suggesting a successful mice model was built. EHLJ7 downgraded the DAI of DSS-induced mice in a dose-dependent manner (**Figure 1D**).

**Table 2:** Disease activity index scoring standard.

Score	Weight loss percentage (％)	Stool trait	Degree of blood
0	0	Normal	Normal
1	1~	Loose (+)	Hidden blood (+)
2	5~	Loose (++)	Hidden blood (++)
3	10~	Diarrhea (+)	Blood stools (+)
4	>15	Diarrhea (++)	Blood stools (++)

### EHLJ7 Attenuates Colonic Pathological Damage in UC Mice

**Figure 2:**
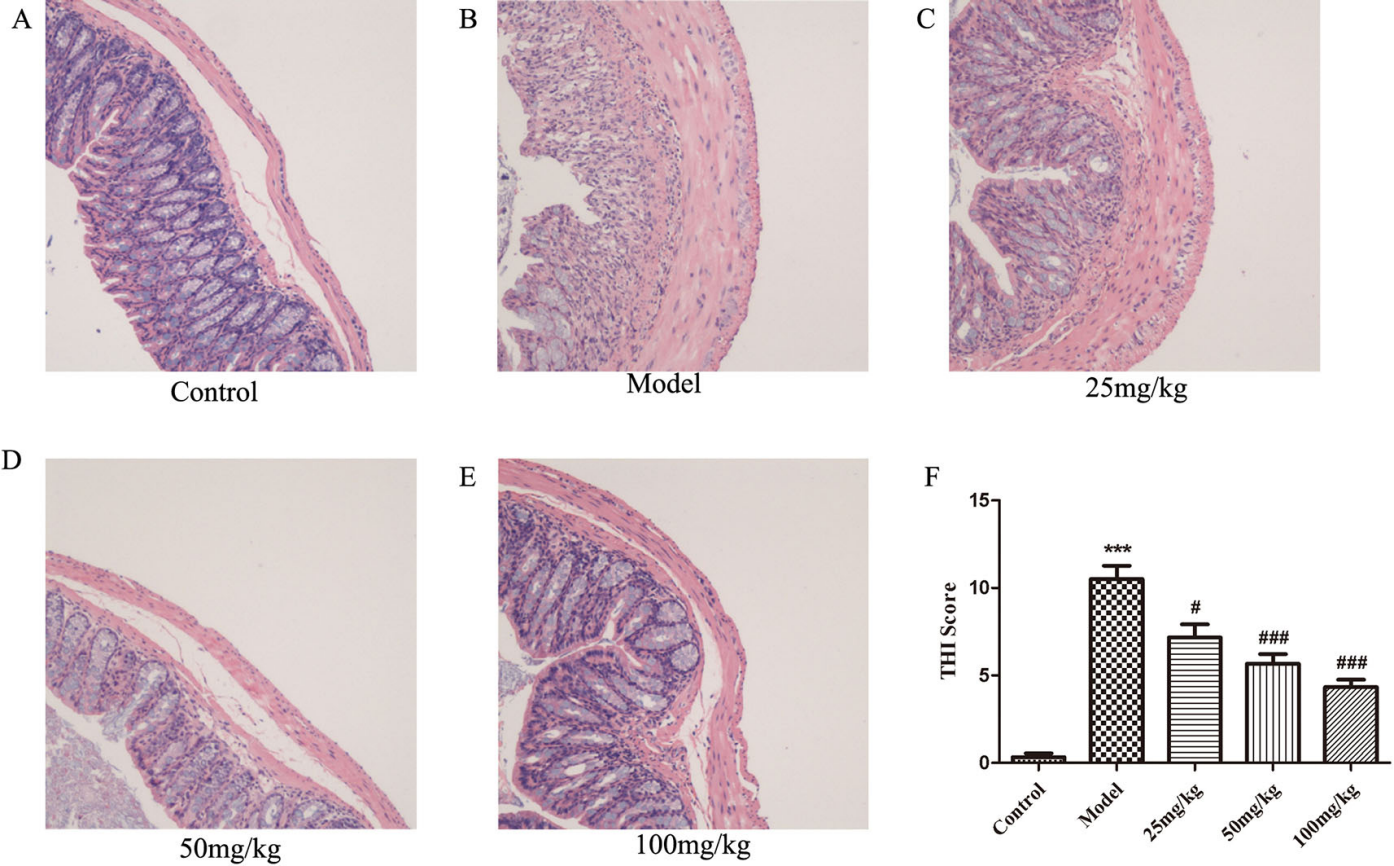
The effect of EHLJ7 on pathological damage to colon tissue. **(A)** HE staining for colon tissue of control group; **(B)** H&E staining for colon tissue of DSS-induced UC group; **(C–E)** HE staining for colon tissue of EHLJ7 treatment group (25, 50, and 100 mg/kg respectively). **(F)** Result of tissue histopathological index (THI) of colon. EHLJ7 (25, 50, and 100 mg/kg) could relieve DSS-induced colon damage and reduce histopathological index in the colons of mice with UC. ***P < 0.001 compared with control group; ^#^P< 0.05, ^###^P < 0.001 compared with DSS model group. Mean values ± SEM are shown. n = 6.

Observation of H&E-stained sections showed that the pathological damage and histological scores of the colon caused by DSS were significantly improved in all EHLJ7 treated DSS-induced mice (**Figures 2A–E**). Colonic mucosa was obviously shed in DSS-induced colon sections. A large number of inflammatory cells, mainly lymphocytes and neutrophils, infiltrated in the inflammatory lesions. Inflammatory fibrous tissue hyperplasia could be seen at the bottom of the ulcer. Moreover, EHLJ7 significantly improved DSS induced tissue histological index (THI), which reflects the damage of colon tissues (**Figure 2F**). THI analysis results showed that the edema, inflammation and infiltration of mucosal layer, submucosa, and muscle layer were greatly improved by EHLJ7.

### EHLJ7 Increases the Production of Butyric Acid in Feces of UC Mice *In Vivo*

**Figure 3:**
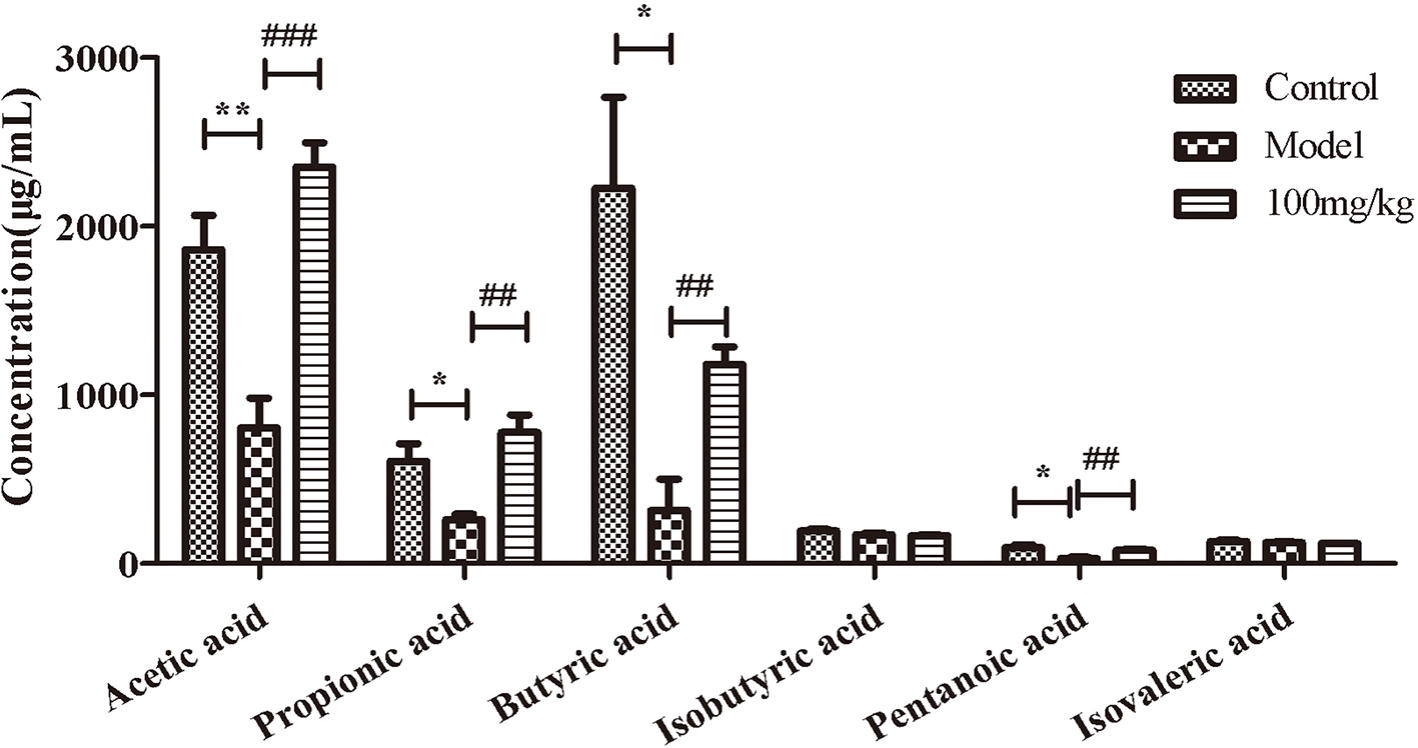
The effect of EHLJ7 on the production of SCFAs in UC-induced mice. EHLJ7 could significantly increase the content of acetic acid, propionic acid, butyric acid and pentanoic acid in the feces compared with the model group.*P < 0.05, **P < 0.01 compared with control group; ^##^P < 0.01, ^###^P < 0.001 compared with DSS model group. Mean values ± SEM are shown. n = 6.

We further explored the influence of EHLJ7 on the production of SCFAs in feces of UC mice *in vivo*. Results showed that EHLJ7 could significantly increase the production of SCFAs in DSS-induced mice (**Figure 3**). The contents of acetic acid, propionic acid, butyric acid, and pentanoic acid in the feces of the model group were significantly lower than those of the control group, while the production of SCFAs in the feces of mice administrated with EHLJ7 (100 mg/kg) was significantly increased. The contents of pentanoic acid and isovaleric acid did not change much.

### EHLJ7 Increases the Production of Butyric Acid in Feces of Mice *In Vitro*

**Figure 4:**
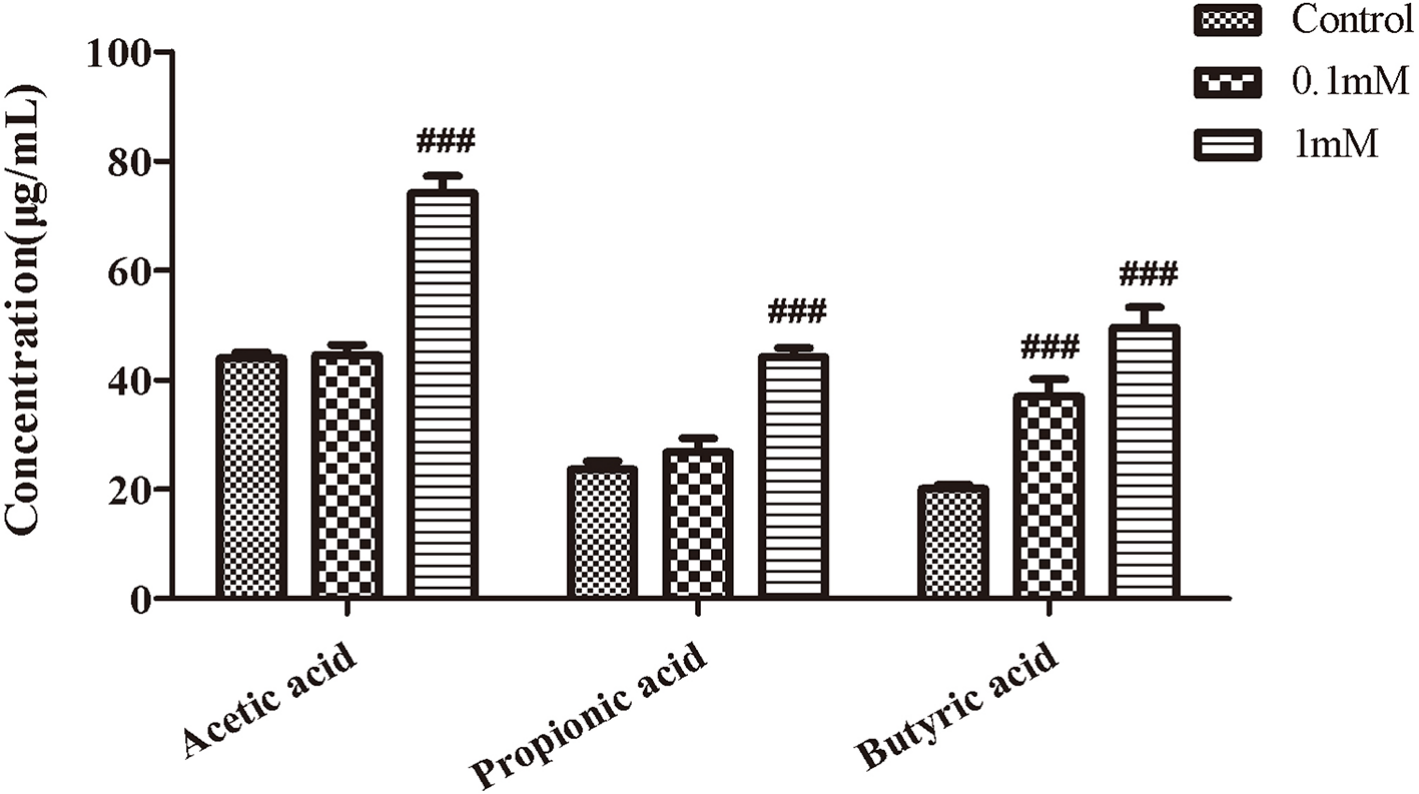
The effect of EHLJ7 on the secretion of SCFAs *in vitro*. After incubated with feces from normal mice for 24 h, EHLJ7(1 mM) significantly increased the production of acetic acid, propionic acid, and butyric acid.^###^P < 0.01 compared with DSS model group. Mean values ± SEM are shown. The experiment was repeated three times. n = 3.

We next observed the effect of EHLJ7 on the production of SCFAs in feces of mice *in vitro*. Results showed that EHLJ7 could significantly increase the production of SCFAs in feces of mice *in vitro* (**Figure 4**). EHLJ7 (1 mM) significantly increased the production of acetic acid, propionic acid, and butyric acid after incubation with feces from normal mice for 24 h, consistent with the *in vivo* results.

### EHLJ7 Reduces the Expression of p-JAK2, p-STAT3, and SOCS1 and Levels of TNFα and IL-6 in Colon Tissue

**Figure 5:**
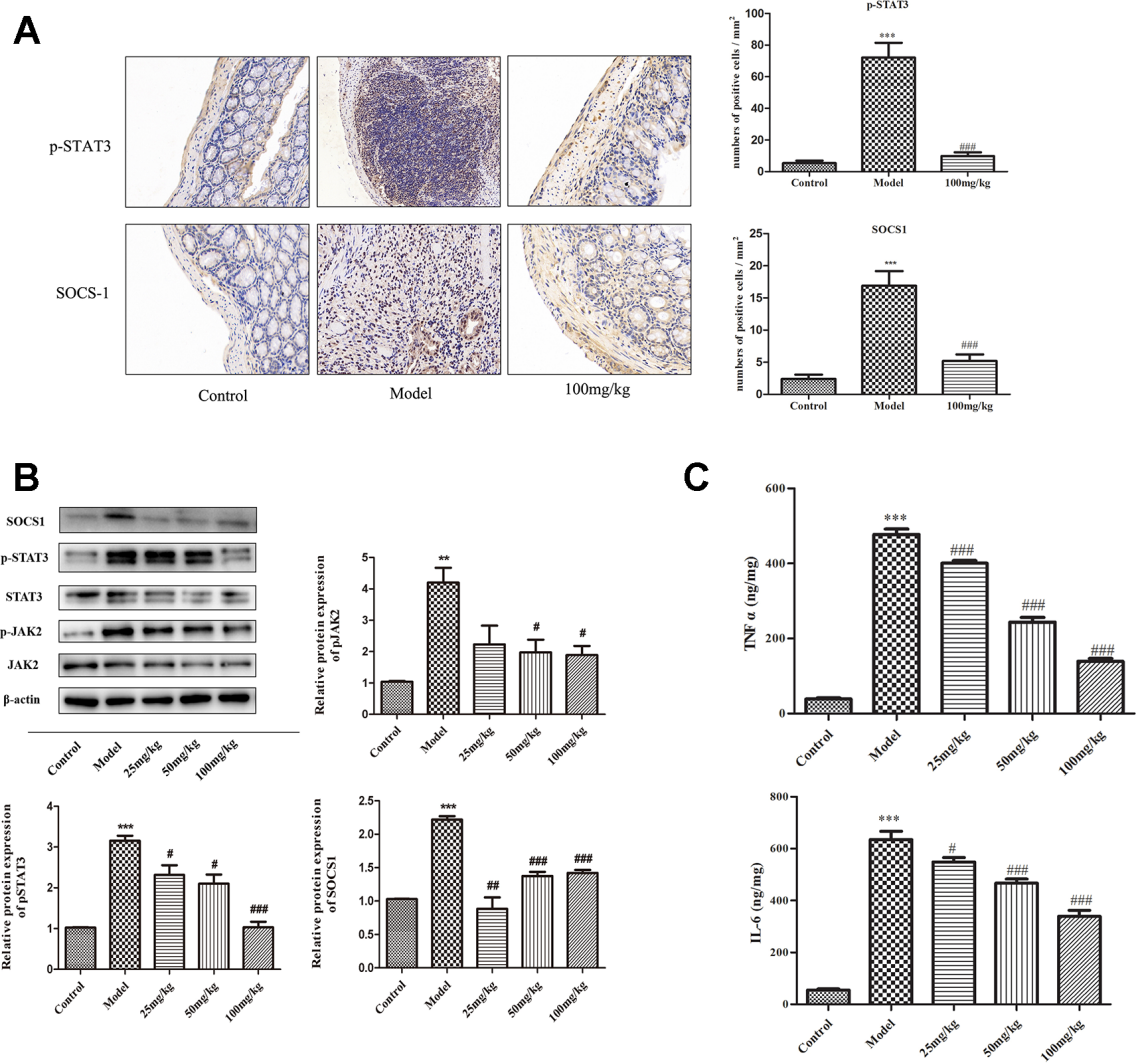
Inhibitory effect of EHLJ7 on expression of JAK2/STAT3/SOCS1 pathway and inflammatory cytokines in DSS induced UC mice. **(A)** IHC staining of the expression of p-STAT3 and SOCS1. EHLJ7 (100 mg/kg) could inhibit the expression of p-STAT3 and SOCS1. **(B)** Western-blot results and corresponding quantitative analysis in mouse colon tissue. EHLJ7 (50 and 100 mg/kg) effectively decreased JAK2 expression compared with the DSS model group. Treatment with EHLJ7 (25, 50, and 100 mg/kg) inhibited STAT3 and SOCS1 expression. **(C)** The inhibitory effect of EHLJ7 on levels of TNFα and IL-6. **P < 0.01, ***P < 0.001 compared with control group; ^#^P < 0.05, ^##^P < 0.01 and ^###^P < 0.001 compared with DSS model group. Mean values SEM are shown. n = 6.

It is known that STAT3 phosphorylation is induced by JAK2, resulting in increased transcription of the target genes including SOCS1. Results of immunohistochemistry (IHC) staining showed that the expressions of p-STAT3 and SOCS1 were significantly increased in DSS group mice and reduced in 100 mg/kg EHLJ7 administered mice (**Figure 5A**). Western blot analysis showed that EHLJ7 could inhibit the expression of p-JAK2, p-STAT3 and SOCS1 *in vivo* (**Figure 5B**), consistent with IHC results.

Pro-inflammatory cytokines including of TNFα and IL-6 play important role in the pathogenesis of UC. IL-6 also reported to activate the JAK/STAT pathway. ELISA results showed that EHLJ7 could inhibit the expression of TNFα and IL-6 *in vivo* (**Figure 5C**).

### Both Butyric Acid and EHLJ7 Inhibited Activation of JAK2/STAT3/SOCS1 Signaling Pathway *In Vitro*

**Figure 6:**
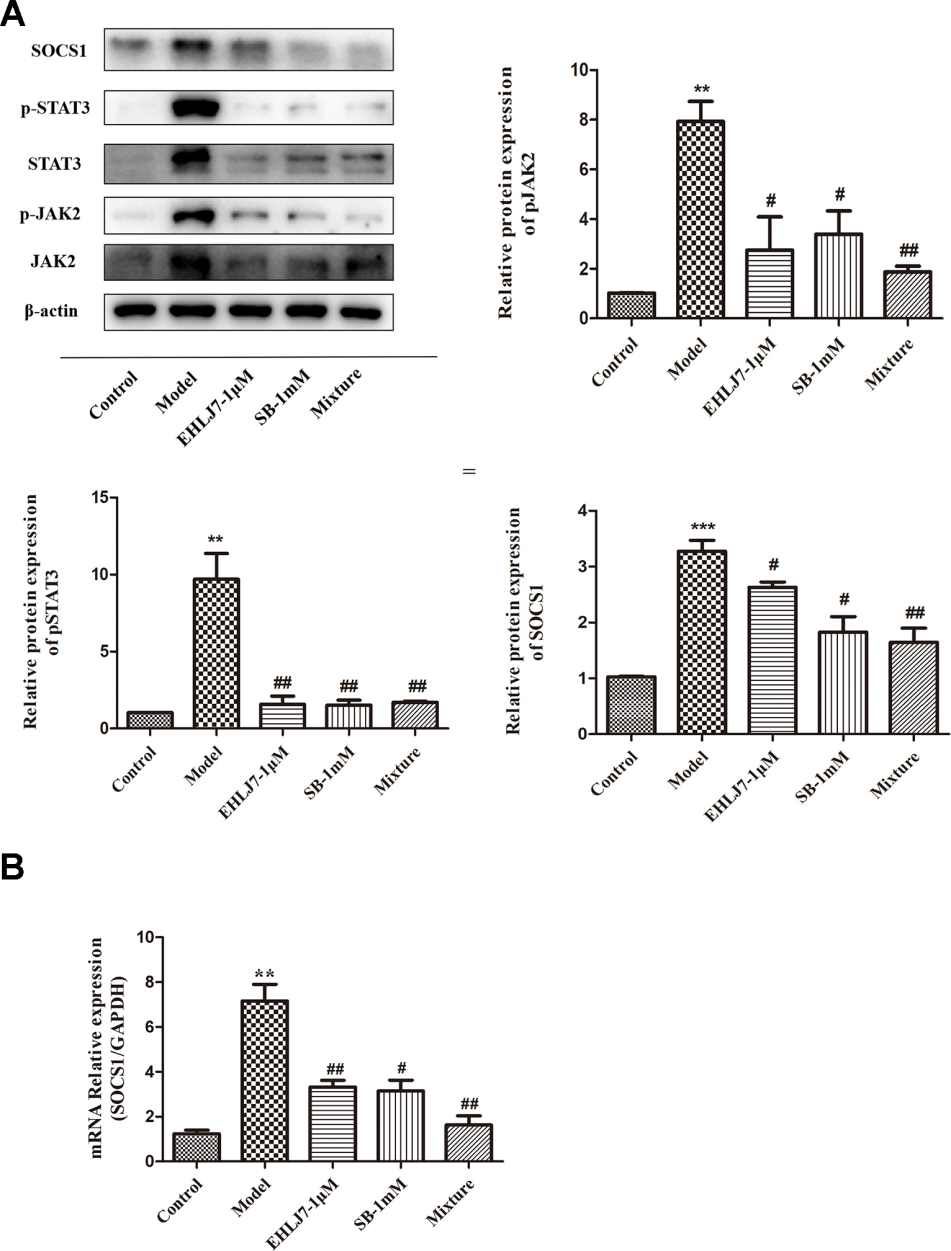
Increasing inhibitory effect of EHLJ7 with sodium butyrate on expression of JAK2, STAT3 and SOCS1 IL6-stimulated IEC6 cell assays *in vitro*. **(A)** Western-blot results of the expression of JAK, STAT3 and SOCS1. Sodium butyrate (1 mM) could strength the inhibition effect of EHLJ7 on the JAK2/STAT3 pathway, especially on SOCS1 expression in IEC6 cells stimulated with 25 ng/mL IL-6 for 24 hours. **(B)** qPCR results of SOCS1 in IEC6 cells. EHLJ7 also inhibited the gene expression of SOCS1. **P < 0.01, ***P < 0.001 compared with control group; ^#^P < 0.05, ^##^P < 0.01compared with IL-6 stimulant. Mean values SEM are shown. n = 3.

To further explore the therapeutic effects of EHLJ7 and butyric acid, we developed IL6-stimulated IEC6 cells assay *in vitro*. Results showed that EHLJ7 could inhibit the JAK2/STAT3/SOCS1 signaling pathway and sodium butyrate could increase the inhibition effect of EHLJ7 (**Figure 6A**). After stimulating IEC6 cells with 25 ng/ml IL-6 for 24 h, the expression of p-JAK2, p-STAT3, and SOCS1 significantly increased. Both the sodium butyrate (1 mM) administered group and the EHLJ7 administered group (1 μM) alone showed a reduced expression of pSTAT3 and SOCS1. EHLJ7 with addition of sodium butyrate could also inhibit the JAK2/STAT3/SOCS1 pathway, especially on SOCS1. Consistently, qPCR analysis also showed that EHLJ7 had inhibitory effect on the activation of IL6-stimulated SOCS1 (**Figure 6B**).

## Discussion

Ulcerative colitis is a chronic non-specific inflammatory disease that invades in the colonic mucosa (Zheng et al., 2017). The lesions are mostly located in the colon and rectum (Kovach et al., 2019). UC is recurrent and easy to be cancerous (Hanaoka et al., 2018; Ganesananthan et al., 2019). However, there are no good therapeutic drugs in clinic so far.

Coptidis is reported to have anti-ulcerative colitis activity (Krzystek-Korpacka et al., 2017; Zhu et al., 2019). In this study, we have made a new exploration of the anti-UC activity and mechanism of a novel monomeric compound EHLJ7. We first examined the therapeutic effect of EHLJ on DSS-induced UC mice. The results showed that EHLJ7 could dose-dependently improve the body weight loss, colonic contracture, and the elevated DAI score in mice induced by UC. Histological pathological results showed that EHLJ7 (100 mg/kg) significantly decreased inflammatory infiltration, edema and the exudation of eosinophils and neutrophils induced by DSS, which illustrated that EHLJ7 had a good therapeutic effect on mice with ulcerative colitis.

SCFAs are the major products from commensal bacterial fermentation of indigestible dietary fibers in the cecum and colon by gut microbes. The principal SCFAs in the intestine include acetate, propionate, and butyrate. Acetate and propionate exist in both small and large intestines, while butyrate is produced mainly in the colon and cecum. SCFAs are absorbed by passive diffusion or carrier-mediated transportation into portal vein, dispersed to peripheral tissues to affect metabolism and the function of peripheral tissues such as inhibiting inflammation in colon (28, Ratajczak et al., 2019). In our study, we examined the expression of short-chain fatty acids in the intestinal contents of mice in each experimental group. Results showed that the expression of acetic acid, propionic acid, butyric acid, and pentanoic acid in the intestinal tract of DSS mice were significantly reduced compared with the control group, while the expression of butyric acid and pentanoic acid were increased in EHLJ7 treated group (100 mg/kg) and the expression of acetic acid and propionic acid were even higher than the control group with EHLJ7 administration. In addition, the co-incubation of EHLJ7 and mouse intestinal contents for 24 h *in vitro* showed that EHLJ7 could also increase the expression of short-chain fatty acids in feces. These results revealed that EHLJ7 could improve the metabolic disorder of intestinal flora to a certain extent.

Janus Kinase 2 (JAK2) is a non-receptor tyrosine protein which is widely distributed in various tissues and cells and can be activated by various cytokines. The signal transducer and activator of transcription (STAT)-3 is an important transcription factor involved in the inflammatory pathogenesis of colitis. Suppressor of cytokine signaling-1 (SOCS1) is a crucial inhibitor of cytokine signaling and could inhibit inflammatory signaling (Kimura et al., 2005). Specially, the JAK/STAT pathway has been reported to be most strongly tyrosine phosphorylated in human ulcerative colitis and Crohn’s disease patients as well as in dextran sulfate sodium (DSS)-induced colitis in mice. To further explore the relationship among SCFAs, JAK2, STAT3, and SOCS1 and the development of ulcerative colitis, we initially observed changes in inflammation-related indicators *in vivo*. IHC results showed that STAT3 and SOCS1 were highly expressed in the colon of DSS mice and were significantly down-regulated in the colon of EHLJ7 (100 mg/kg) treated mice. Simultaneously, Western blot analysis showed that EHLJ7 had a tendency to decrease the expression of pJAK2, pSTAT3, and SOCS1 in the colon of DSS mice. On this basis, we want to further explore the effect of butyrate on JAK2/STAT3/SOCS1 signaling pathway. Zhou L et al. reported that butyrate-IL6-JAK2-STAT3 pathway could be an effective novel approach in the treatment of inflammatory disease (Zhou et al., 2018). EHLJ7 could greatly inhibit the levels of TNFα and IL-6 in colon tissues of DSS-induced mice. Western blot analysis and qPCR analysis in IEC6 cells stimulated by IL-6 *in vitro* revealed that EHLJ7 could cooperate with butyrate to exert its anti-ulcerative colitis effect by inhibiting the activation of JAK2/STAT3/SOCS1 pathway.

In summary, we demonstrated that EHLJ7 have good effects on DSS-induced colitis. EHLJ7 may induce intestinal flora to overexpress SCFA, especially butyric acid and then inhibit colitis inflammation through inhibition of JAK2/STAT3/SOCS1 pathway. Therefore, we expect EHLJ7 will be developed as a possible candidate for the treatment of colitis.

## Data Availability Statement

All datasets for this study are included in the article.

## Ethics Statement

The animal study was reviewed and approved by the Institutional Animal Care and Use Committee, Chinese Academy of Medical Sciences, China. Written informed consent was obtained from the owners for the participation of their animals in this study.

## Author Contributions

XT and XL contributed study design, data collection, and data analysis. YW and XT contributed data collection. ZZ and AD contributed compound synthesis. ZZ also reviewed and agreed the paper. WW contributed literature search. HZ, LW, and HQ contributed final manuscript version and Funds Collection. All authors reviewed the manuscript.

## Funding

This work was financially supported by the Drug Innovation Major Project (2018ZX09711001-002-002 and 2018ZX09711001-003-001), the Fundamental Research Funds for the Central Universities (3332019073), CAMS Innovation Fund for Medical Sciences (2016-12M-3-011), and the Opening Funds for State Key Laboratory of Bioactive Substances and Functions of Natural Medicines (GTZK201906). There is no conflict to declare regarding funding.

## Conflict of Interest

The authors declare that the research was conducted in the absence of any commercial or financial relationships that could be construed as a potential conflict of interest.
